# Very Late Stent Thrombosis 42 Months after Implantation of Sirolimus-Eluting Stent and Discontinuation of Antiplatelet Therapy

**DOI:** 10.1155/2009/713292

**Published:** 2009-03-25

**Authors:** Dirk Sibbing, Karl-Ludwig Laugwitz, Lorenz Bott-Flügel, Jürgen Pache

**Affiliations:** Department of Cardiology, Deutsches Herzzentrum München & 1. Medizinische Klinik, Technische Universität München, Lazarettstrasse 36, 80636 München, Germany

## Abstract

Although safety profiles of sirolimus-eluting stents do not seem to differ in short-to-medium term from those of bare-metal stents, late stent thrombosis after deployment of drug-eluting stents has emerged as a potential safety concern in the era of high-pressure stent implantation. Here, we describe the case of a patient with acute myocardial infarction due to stent thrombosis of a sirolimus-eluting stent 42 months after stent deployment and 5 weeks after discontinuation of aspirin treatment. To the best of our knowledge, this is one of the most delayed cases of sirolimus-eluting stent thrombosis described so far. The case emphasizes the potential risk that late stent thrombosis can unpredictably occur at any time point after drug-eluting stent deployment.

## 1. Introduction

Stent thrombosis following percutaneous coronary intervention (PCI) is regarded as a complication of the procedure mainly occurring during the first 30 postprocedural days. However, concerns have been raised that late stent thrombosis (>30 days after PCI)—basically due to delayed endothelialization of the stent struts—may be a potential limitation following deployment of drug-eluting stents (DESs) [1]. Premature discontinuation of antiplatelet treatment was identified as the most important independent predictor of stent thrombosis after successful DES implantation [1]. Recently, data are accumulating that late or very late (>1 year after PCI) stent thrombosis can unpredictably occur at any time point after DES deployment [2–5]. In this case report, we describe a patient with acute myocardial infarction due to stent thrombosis of a sirolimus-eluting stent 42 months after stent deployment and 5 weeks after discontinuation of aspirin treatment.

## 2. Case Presentation

**Figure 1:**
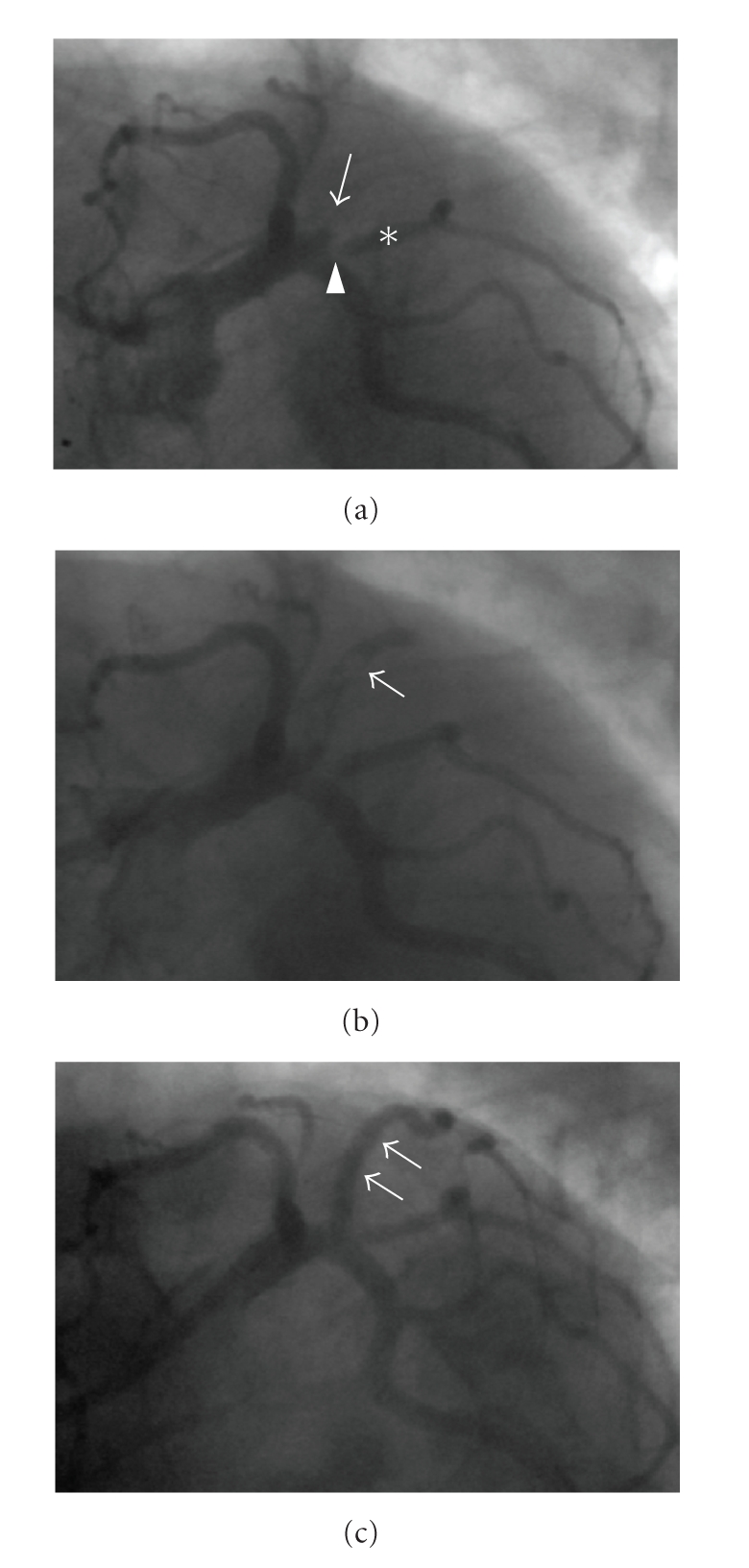
(a) Coronary angiography showing a total occlusion of the 1st left posterolateral branch of the left circumflex artery (see arrow), an ostial stenosis (see arrow head) of the 2nd left posterolateral branch (proximal to the bare-metal stent), and a patent bare-metal BeStent (see asterisk). (b) Coronary angiography demonstrating stent occlusion of sirolimus-eluting stent and extensive thrombus after guidewire passage (see arrow). (c) Coronary angiography documenting vessel patency after balloon angioplasty.

 A 72-year-old man with a known history of coronary artery disease (CAD) was admitted to our cardiology department in September 2006 with symptoms of acute and ongoing chest pain at rest. 42 months before this admission, in March 2003, coronary angiography showed a two-vessel coronary disease with two significant lesions of the left circumflex (LCx) artery. One sirolimus-eluting stent (Cypher, 3 mm diameter, 13 mm long; Cordis, Miami Lakes, Fla, USA) was placed in the 1st left posterolateral branch and a second bare-metal stent (BeStent, 2.5 mm diameter, 12 mm long, Medtronic, Minneapolis, Minn, USA) was implanted in the 2nd left posterolateral branch of the LCx. The patient was discharged on dual antithrombotic medication consisting of aspirin and clopidogrel. In September 2003, 6 months after PCI, coronary angiography demonstrated both a patent bare-metal and sirolimus-eluting stents. Clopidogrel was discontinued another 6 months later. Henceforth, the patient was on monotherapy with aspirin 200 mg/d. The following three years were uneventful regarding his CAD. At the end of July 2006, 5 weeks before his present admission, aspirin was discontinued due to planned dental surgery and planned coloscopy with a potential demand for polypectomy. On his present admission, emergency coronary angiography was performed due to ongoing chest pain, a positive troponin T (0.21 ng/mL) and an elevated level (350 IU/L) of creatine kinase (CK). Angiography showed total occlusion of the 1st left posterolateral branch, an ostial stenosis of the 2nd left posterolateral branch (proximal to the bare-metal stent) and a patent bare-metal BeStent (Figure 1(a)). Further on, angiography demonstrated stent occlusion of sirolimus-eluting stent and extensive thrombus after guidewire passage (Figure 1(b)). Ballon angioplasty was successful and restored vessel patency (Figure 1(c)); peak concentration of CK was 2116 IU/L.

## 3. Discussion

To the best of our knowledge, this is one of the most delayed cases of sirolimus-eluting stent thrombosis described so far. Late-stent thrombosis, 42 months after deployment, was probably due to aspirin withdrawal accompanied by a lack of endothelialization of the stent struts. A second possible and previously reported mechanism could be persistent and extensive inflammatory reaction of the arterial wall surrounding the stent as a reaction to the polymer [6]. The case emphasizes the potential risk that late stent thrombosis can unpredictably occur at any time point after drug-eluting stent deployment [7]. Stent thrombosis must be considered as a potential risk when discontinuation of antiplatelet medication is contemplated and any decision to stop antiplatelet therapy for whatever reason in patients exhibiting drug-eluting stents should take this into account. Moreover, this case and others raise questions about current recommendations for the duration and indications to discontinue antiplatelet therapy after DES implantation.
